# Dual plating or dual plating combined with compression bolts for bicondylar tibial plateau fractures: a retrospective comparative study

**DOI:** 10.1038/s41598-021-87510-6

**Published:** 2021-04-08

**Authors:** Zhongzheng Wang, Yuchuan Wang, Siyu Tian, Zhanchao Tan, Xiangtian Deng, Kuo Zhao, Zhanle Zheng, Wei Chen, Zhiyong Hou, Yingze Zhang

**Affiliations:** 1 Department of Orthopaedic Surgery, the Third Hospital of Hebei Medical University, Shijiazhuang, Hebei 050051 People’s Republic of China; 2 Key Laboratory of Biomechanics of Hebei Province, Shijiazhuang, Hebei 050051 People’s Republic of China; 3 School of Medicine, Nankai University, Tianjin, 300071 People’s Republic of China; 4 NHC Key Laboratory of Intelligent Orthopaedic Equipment, Shijiazhuang, Hebei 050051 People’s Republic of China

**Keywords:** Diseases, Trauma, Outcomes research, Clinical trial design, Fracture repair, Therapeutics

## Abstract

The aim of this study was to compare secondary loss of reduction outcomes in dual plating fixation and dual plating combined with compression bolt fixation for bicondylar tibial plateau fractures (TPFs). We performed a retrospective study from January 2015 to April 2019. A consecutive series of 72 bicondylar TPFs underwent surgical treatment and was divided into two groups: group 1 (dual plating, n = 46) and group 2 (dual plating combined with compression bolts, n = 26). The outcomes collected included demographic characteristics, imaging characteristics, intraoperative indicators, clinical outcomes and reduction quality after surgery. Functional outcome was rated according to the Hospital for Special Surgery (HSS) score and Lysholm score. The secondary loss of reduction rate in group 2 was reduced compared with that in group 1 (*P* = 0.025), and the mean HSS score of group 2 was higher than that of group 1 (*P* = 0.013). The rate of complications was 30.4% (14/46) in group 1 and 30.8% (8/26) in group 2 (*P* = 0.976). Compared with single dual plating fixation, dual plating combined with compressing bolt fixation reduced the secondary loss of reduction rate for patients with bicondylar TPFs and showed better functional outcomes.

## Introduction

Tibial plateau fractures (TPFs) are one of the most challenging injuries in orthopaedic trauma and usually require surgical treatment. Bicondylar TPFs are often defined as complex fractures, which are described as type V and VI by the Schatzker classification system and as 41-type C1-3 by the AO/Orthopaedic Trauma Association classification^1–4^. In recent decades, several therapeutic strategies have been proposed by different authors for the treatment of bicondylar TPFs. In general, the vast majority of bicondylar TPFs require internal fixation, such as lateral locking plating or dual medial and lateral plating fixation, and additional posteromedial buttress plating if necessary^5–8^. External fixation is mostly used in patients with open TPFs or severe soft tissue injury around the knee^7,9–11^.

In recent years, the secondary loss of reduction after surgery for TPFs has become a hot topics among orthopaedic surgeons^7,12,13^. Although several authors have studied the clinical outcomes and prognosis of surgical management for TPFs, the reasons for the loss of secondary reduction after surgery are still controversial. The study of Ali et al.^13^ suggested that the secondary loss of reduction might be associated with earlier weight-bearing exercise, greater preoperative displacement, more comminuted fracture fragmentation and severe osteoporosis. Subsequently, Weaver et al.^7^ reported that the fracture pattern and fixation type were also associated with a high reduction loss rate. Compared with single later-locked plating, dual plating fixation for the treatment of bicondylar TPFs with medial coronal fracture can significantly reduce the secondary loss of reduction rate. However, Kim et al.^12^ found that the secondary reduction loss rate in bicondylar TPFs with medial coronal fractures treated with dual plate fixation was as high as 48.5%, which may be caused by different definitions of reduction loss and the differences in the subjects.

Recently, Zhang et al.^14^ found that compression bolts can effectively prevent the loss of fracture reduction, and were the first to apply them in the treatment of TPFs and calcaneal fractures. Later, the author carried out a series of clinical studies using compression bolts and obtained satisfactory prognostic results^14,15^. To the best of our knowledge, few studies have assessed the secondary loss of reduction in bicondylar TPFs treated with dual plating combined with compression bolts. Therefore, the author hypothesized that the application of compression bolts during the operation could improve the secondary loss of the reduction rate.

## Results

In this study, 71 patients (72 knees, left 35 and right 37) with bicondylar TPFs were enrolled in more than 4 years, including 52 men and 20 women. The mean age was 47.0 years (range: 22–72 years). All bicondylar TPFs were divided into two groups according to the internal fixation method used. Group 1 contained 46 cases (63.9%), which were treated with dual plating. Group 2 consisted of 26 cases (36.1%), which were treated with dual plating combined with compression bolts. The demographic data and imaging characteristics of the two groups were not significantly different in this study (Table 1).

**Table 1:** Demographic and imaging characteristics of two groups.

Variables	Group 1 (n = 46) Mean ± SD/n, (%)	Group 2 (n = 26) Mean ± SD/n, (%)	*P* value
Age (yrs)	46.9 ± 12.3	47.2 ± 12.5	0.926
**Gender**			0.903
Male	33 (71.7)	19 (73.1)	
Female	13 (28.3)	7 (26.9)	
BMI (Kg/m^2)	25.6 ± 3.7	26.9 ± 3.7	0.153
**Affected side**			0.074
Left	26 (56.5)	9 (34.6)	
Right	20 (43.5)	17 (65.4)	
Smoking (yes)	11 (23.9)	7 (26.9)	0.777
Alcoholism (yes)	13 (28.3)	9 (34.6)	0.574
Hypertension (yes)	7 (15.2)	3 (11.5)	0.665
Diabetes (yes)	6 (13.0)	2 (7.7)	0.488
**Living area**			0.526
Rural	30 (65.2)	15 (57.7)	
Urban	16 (34.8)	11 (42.3)	
**Schatzker classification**			0.163
Type V	20 (43.5)	7 (26.9)	
Type VI	26 (56.5)	19 (73.1)	
**OTA/AO classification**			0.240
41-C1	8 (17.4)	2 (7.7)	
41-C2	17 (37.0)	7 (27.0)	
41-C3	21 (45.6)	17 (65.3)	
Coronal fracture (yes)	20 (43.5)	13 (50.0)	0.594
Comminution (yes)	31 (67.4)	19 (73.1)	0.615
Time to surgery (days)	9.5 ± 4.7	9.5 ± 3.8	0.968
Follow-up time (months)	18.2 ± 6.0	17.2 ± 5.6	0.479

Table 2 shows that the number of cases with secondary reduction loss was 21 (45.7%) in group 1 and 5 (19.2%) in group 2 (*P* = 0.025). There were 15 knees (32.6%) in group 1 and 3 knees (11.5%) in group 2, and the difference in plateau widening was greater than 5 mm at the final follow-up compared to the immediate postoperative radiographs (*P* = 0.047). There were 9 knees (19.6%) in group 1 and 0 knees (0.0%) in group 2, and the difference in plateau articular step-off was greater than 3 mm at the final follow-up compared to the immediate postoperative radiographs (*P* = 0.016). In addition, the mean HSS score of group 1 was 88.74 ± 5.70, which was lower than that of group 2 (91.69 ± 4.02, *P* = 0.013). No significant differences in the operative time, intraoperative blood loss, anaesthetization method, bone grafting, hospital stay, mean time of fracture healing, rate of immediate postoperative reduction loss or Lysholm score were noted between the two groups.

**Table 2:** Clinical results of two groups.

Variables	Group 1 (n = 46) Mean ± SD/n, (%)	Group 2 (n = 26) Mean ± SD/n, (%)	*P* value
**Operative time (minutes)**			0.725
1–180	37 (80.4)	20 (76.9)	
> 180	9 (19.6)	6 (23.1)	
**Intraoperative blood loss (ml)**			0.632
1–400	33 (71.7)	20 (76.9)	
> 400	13 (28.3)	6 (23.1)	
**Anesthetization**			0.442
Intraspinal	22 (47.8)	10 (38.5)	
General	24 (52.2)	16 (61.5)	
**American Society of Anesthesiologists class**			0.801
I	8 (17.4)	3 (11.5)	
II	30 (65.2)	18 (69.2)	
III or above	8 (17.4)	5 (19.3)	
Bone grafting (yes)	15 (32.6)	9 (34.6)	0.862
Hospital stay (days)	21.8 ± 10.8	21.4 ± 13.0	0.906
Mean fracture healing time (months)	4.6 ± 1.8	4.5 ± 2.1	0.721
Loss of immediate postoperative reduction	7 (15.2)	4 (15.4)	0.985
Secondary loss of reduction	21 (45.7)	5 (19.2)	0.025
**Plateau widening (mm)**			0.047
1–5 reference	31 (67.4)	23 (88.5)	
> 5	15 (32.6)	3 (11.5)	
**Articular step-off (mm)**			0.016
1–3 reference	37 (80.4)	26 (100.0)	
> 3	9 (19.6)	- (0.0)	
**TPA (degree)**			0.919
1–5 reference	44 (95.7)	25(96.2)	
> 5	2 (4.3)	1 (3.8)	
**PAM (degree)**			0.124
1–5 reference	36 (78.3)	24 (92.3)	
> 5	10 (21.7)	2 (7.7)	
HSS score	88.74 ± 5.70	91.69 ± 4.02	0.013
Lysholm score	83.15 ± 6.40	83.42 ± 5.06	0.854

**Figure 1:**
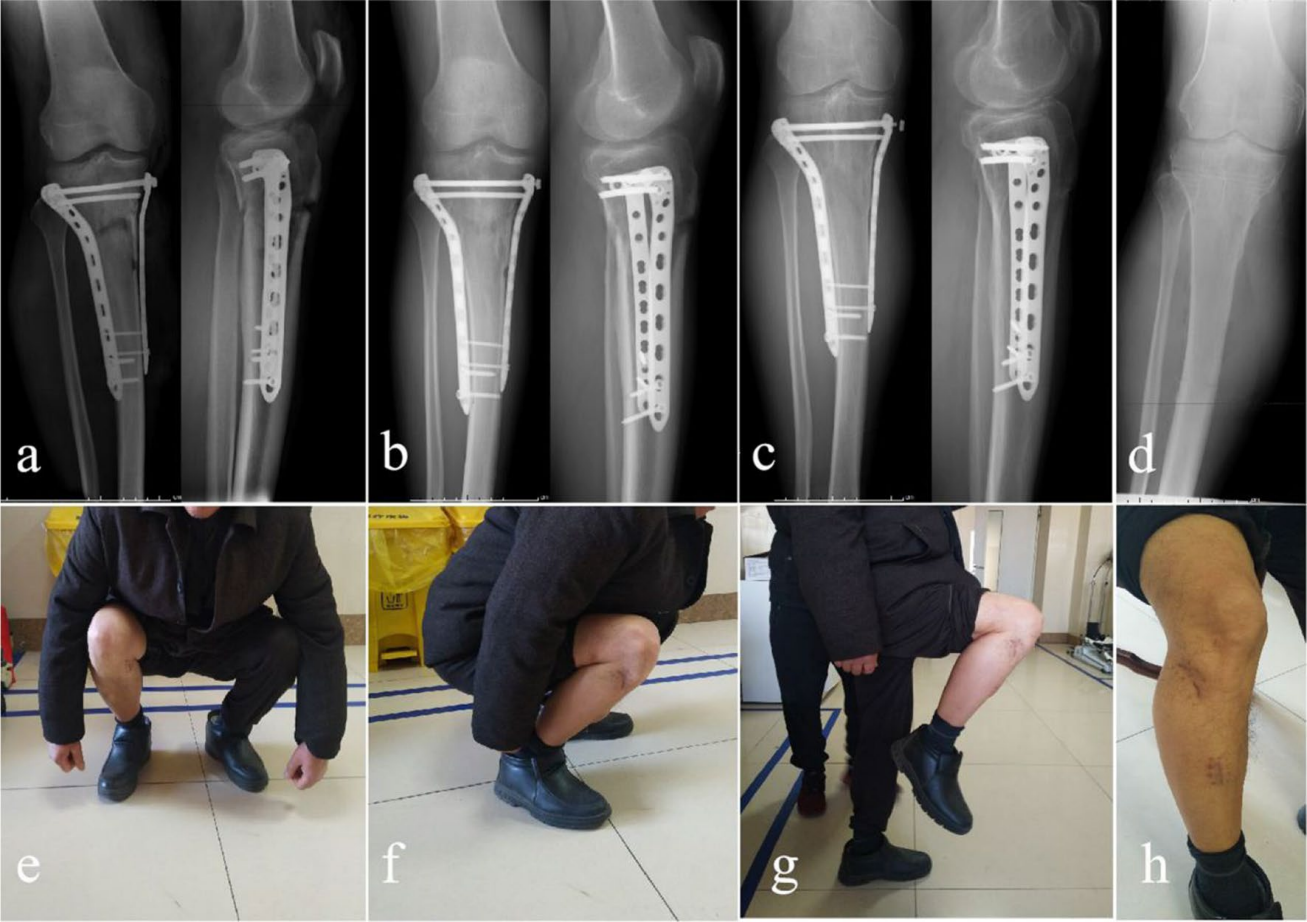
Case example of a patient treated with dual plating combined with compression bolt. (**a**) Immediate post-operative films (AP and lateral views). (**b**) 12 months post-operative films (AP and lateral views). (**c**) 19 months post-operative films (AP and lateral views). (**d**) postoperative films (AP view) after removing the internal fixation. (**e**–**h**) Functional posture and incision images one year follow-up after operation. We have obtained the patient's informed consent to publish these images. In addition, we have deleted all personal information to protect the patient's privacy.

All patients recovered well in this study, and no fracture nonunion occurred in any of the patients. Table 3 shows that the complication rate was 30.4% (14/46) in group 1 and 30.8% (8/26) in group 2 (P = 0.976). In group 1, three cases of superficial wound infections occurred, one case developed deep wound infection, and eleven cases developed DVT postoperatively compared with preoperatively. Two cases of superficial wound infections occurred, seven cases developed DVT compared with preoperatively, and one case of a nut of compression bolt falling off occurred in group 2 (Fig. 1). All the relevant symptoms disappeared after conventional treatment.

**Table 3:** Complications of two groups.

Variables	Group 1 (n = 46) n/n, (%)	Group 2 (n = 26) n/n, (%)
Superficial wound infections	3	2
Deep wound infections	1	-
Nonunion	–	-
DVT	11	7
Loosening of internal fixation	–	1
Complication rate	14 (30.4)	8 (30.8)

## Discussion

Our study highlights the difficulty in treating bicondylar TPFs and the high incidence of postoperative secondary loss of reduction. The incidence of immediate postoperative reduction loss in the two groups was 15.2% and 15.4%, respectively, with no significant difference. However, it was found that the secondary loss of reduction rate in group 2 was significantly lower than that in group 1 (19.2% vs 45.7%, *P* < 0.05). This is especially true in terms of plateau widening and articular step-off. Moreover, the HSS score of knee joint function was significantly higher in the group with compression bolts than in the group without compression blots.

Fractures involving the medial and lateral tibial plateaus concomitantly are usually defined as bicondylar TPFs; they are often caused by high-energy trauma in younger patients or osteoporosis in older adults and accompanied by splitting and widening of the platform and depression of the articular surface, requiring surgical treatment to restore the anatomical structure and effectively fix it^16,17^. However, the treatment of bicondylar TPFs remains controversial. Many scholars have suggested that isolated lateral locking plates, dual plating and additional posterior buttress plating can all be used to treat bicondylar TPFs^6,9,18,19^. At present, although lateral locking plates are widely used in bicondylar TPFs, an additional medial support plate is often required when the fragments of the medial condyle are small, comminuted, osteoporotic, or have coronal fractures^7,20,21^. The standard surgical procedure for bicondylar TPFs might be to employ medial and lateral plating to reconstruct bilateral joint surfaces and to prevent varus knee deformities caused by collapse of the medial column^22^. Some studies have shown that correct alignment of the lower limb line can effectively prevent the development of knee arthrosis and modify the foot loading. According to Wolff's law, anatomical axis and sagittal balance line of the lower limb also affected bone remodeling^23^. Traditional open reduction and internal fixation (ORIF) techniques insert both medial and lateral plates through a midline incision in front of the proximal tibia. This surgical approach involves a long incision and severe soft tissue damage around the knee, leading to an increased infection rate^20,22,24^. Subsequently, to solve the problem of infection, a two-incision approach or a lateral approach locking plate/screw fixation method has gradually been advocated by some orthopaedic surgeons^5,24^.

However, in the literature, there are only a few studies concerning the incidence of secondary reduction loss in bicondylar TPFs treated with dual plating combined with compression bolts. In our clinical practice, we found a special slot-designed compression bolt that had the dual functions of reduction and fixation. The split fracture blocks can be automatically reset by rotary compression of the nut. By increasing the transverse pressure, the fracture fragments are closely connected to maintain the reduction effect^14,15^. In addition, this fixation technology has many other advantages. For example, when the nut is tightened, the compression force directly acts on the dual plating instead of the bone surface, avoiding bone damage. In addition, when the nut is tightened, the pressure generated by the bolts will continue for a long time, which can provide sufficient stability for early activities^25^. Furthermore, the unique slotted design can quickly remove the distal part of the bolt, which saves operation time and decreases the risk of pain and stimulation to soft tissue. Subsequently, we applied this device to clinical practice^15^. During the research process, we found that compression bolts were satisfactory in the treatment of complex calcaneal fractures and bicondylar TPFs^25,26^. In addition, Garnavos et al.^27^ used retrograde nailing combined with “independent” compression bolts to treat type C distal femoral fractures, which could provide sufficient stability for early mobilisation.

In the present study, we found that there was no significant difference in the loss of immediate postoperative reduction between the two groups. The group of treated with dual plating combined with compression bolts effectively reduced the secondary reduction loss rate (from 45.7% to 19.2%) and improved knee joint function (HSS score from 88.7 to 91.7). Even more interesting, the use of compression bolts did not increase the mean time of operation, mean intraoperative blood loss, or postoperative complications. No cases of nonunion occurred in any of the patients in either of the two groups, and there was no difference in the healing time. Because, rigid fixation and correct aligment of tibial plateau were powerful factors in promoting bone healing and improving foot loading^23^. One case of the nut of compression bolt falling off occurred during follow-up in group 2, but this did not cause secondary reduction loss or any discomfort (Fig. 1). The reason may have been widespread movement after fracture healing. To prevent the nut from shifting between the soft tissues, the internal fixation was removed early after fracture healing.

Some limitations of this study must be noted. First, the study included a relatively small number of patients with bicondylar TPFs and a relatively short duration of follow-up (mean time of 17.7 months). Therefore, a number of long-term complications may not have been reported. Second, this study was performed retrospectively. Third, the effect of bone quality on secondary loss of reduction was not evaluated. Most patients did not receive bone density examinations on admission or during follow-up. Finally, only simple radiographs were used to evaluate the loss of reduction during the follow-up. CT can more accurately assess the presence of reduction loss, but it is not realistic to perform follow-up CT for all patients.

## Patients and methods

### Ethics statement

After explanation the nature and purpose of the study, written informed consent was obtained from all subjects for print and online open-access publication of their information and related data.The patient in Fig. 1 agreed by written informed consent to publication of the images shown .This study was approved by the Ethics Committee of the Third Hospital of Hebei Medical University. All methods were carried out in accordance with the guidelines (Helsinki Declaration) for biomedical research. All materials, data and associated protocols in this study are available to readers and the publishing team.

### Patients

We performed a review of 218 patients (226 knees) with bicondylar TPFs treated at our level-I trauma centre over four years (from 1 January 2015 to 31 April 2019). The following inclusion criteria were applied: adult patients (age ≥ 18 years), diagnosis of bicondylar TPFs (Schatzker V or VI, 41-type C1-3 of AO/OTA), patients who received open reduction and dual plating or dual plating combined with compression bolt fixation, time of postoperative follow-up of at least one year, preoperative standard CT scans (sagittal, axial and coronal) and X-ray radiography (lateral and anteroposterior (AP) views), and postoperative follow-up weight bearing knee AP and lateral views were available. Patients younger than 18 years, patients who had no available preoperative CT or postoperative X-ray radiography, patients who received other methods of fixation or conservative treatment, open TPFs, previous or pathological TPFs, and patients who did not complete the one-year follow-up were excluded. In total, 154 cases were excluded after review according to the conditions of this study. In total, 110 cases were treated using other fixation methods, such as external fixation, lateral locking plates, and additional posteromedial buttress plating; 14 cases lacked a preoperative CT scan; 9 cases had previous or pathological TPFs; 21 cases had open TPFs; and 33 cases did not complete one year of follow-up.

**Figure 2:**
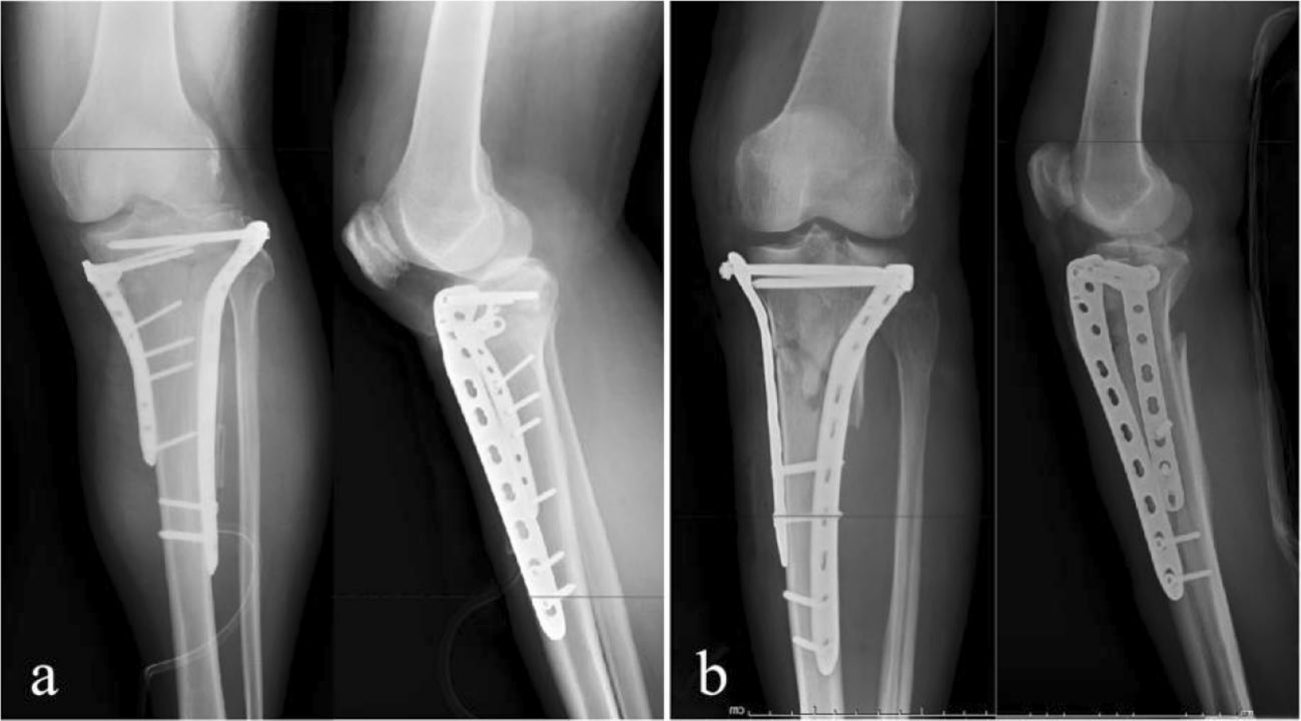
Surgical procedures of dual plating or dual plating combined with compression bolt. (**a**) Immediate post-operative films (AP and lateral views) of patient treated with dual plating. (**b**) Immediate post-operative films (AP and lateral views) of patient treated with dual plating combined with compression bolt.

After reviewing the relevant surgical information, patients treated with dual plating were defined as group 1, and patients treated with dual plating combined with compression bolts were defined as group 2 (Fig. 2).

### Surgical techniques

**Figure 3:**
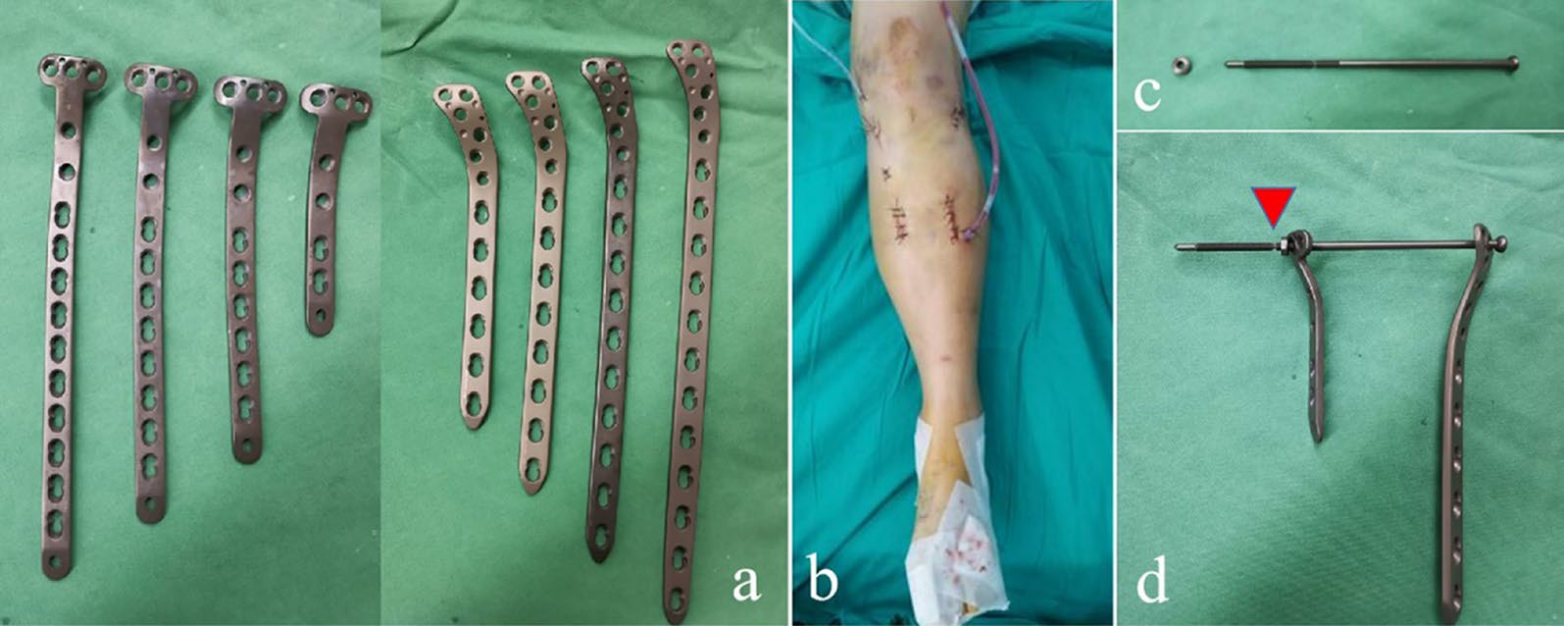
(**a**) Two pre-contoured locking compression plates specially designed for proximal tibia. (**b**) surgical incisions. (**c**) Slot-designed compression bolt. (**d**) The components of dual plating combined with compression bolt were presented. (Red arrow, slot-designed area. Left, medial locking plate. Right, lateral locking plate).

Surgical procedures of the two groups were performed by orthopaedic surgeons from the same team, and the team members had more than 10 years of experience in trauma and orthopaedic surgery. Before surgery, both groups received general anaesthesia or intraspinal anaesthesia and routine antibiotic prophylaxis. For the patients in group 1, dual plate fixation was performed through separate anterolateral and medial skin incisions of the proximal tibia in the supine position, and a universal external fixator or retractor was used for intraoperative reduction. During reduction, the lower limb alignment was corrected, and the width and depression of the tibial plateau were restored. Intraoperative fluoroscopy was used to examine the reduction status. Two pre-contoured locking compression plates (WEGO, Wei Hai city, PR China) specially designed for the proximal tibia were applied to the anterolateral and medial tibia (Fig. 3a). Then, lock screws of suitable length were inserted into the corresponding channels. Intraoperative C-arm fluoroscopy was performed again to confirm the reduction. Layered wound closure and drain insertion were performed after rinsing with normal saline (Fig. 3b).

For the patients in group 2, the incision and reduction technologies were the same as those for the patients in group 1. The difference is that one or two slot-designed compression bolts (WEGO, Wei Hai city, PR China) were applied to replace the original locking screws at the proximal tibia (Fig. 3c).The bolts passed through the corresponding holes of the two platings, and when the nuts passed through the slots, the tails of the bolts could be easily broken off (Fig. 3d). Note that the length of the bolt head to the slot is optional. Intraoperative C-arm fluoroscopy was performed again to confirm the reduction, and close the wound.

### Postoperative management and follow-up

Identical postoperative management schemes were adopted for the two groups. Routine antibiotic treatment was used to prevent wound infection within 24 h after the operation, and routine subcutaneous injection of low-molecular-weight heparin calcium was used to prevent deep vein thrombosis (DVT) of the lower extremities. When the pain subsided, the patients were encouraged to perform non-weight bearing joint movement immediately. According to the general condition and postoperative X-ray examinations, the patients were instructed to perform weight-bearing exercises that usually began approximately 8 weeks after surgery. In addition, load-bearing X-ray examinations (lateral and AP views) were performed at 1, 3, and 6 months after surgery and every 3 months thereafter.

Demographic characteristics, lifestyle risk factors, imaging data of preoperative and postoperative follow-up, information of operation record, the time of fracture healing, postoperative complications, postoperative reduction quality, and functional recovery were collected. All image data of patients with bicondylar TPFs were read by two experienced orthopaedic surgeons. Thus, bicondylar TPFs are classified into types V and VI by the Schatzker classification system, and classified into types 41-C1, 41-C2 and 41-C3 by the AO/OTA classification system^2,3^. The quality of fracture reduction was assessed by two observers based on X-ray (standard AP and lateral radiographs) images taken immediately after surgery and during follow-up. The existence of coronal fractures and comminution were evaluated by preoperative X-ray and CT scans. The functional outcome of the knee was evaluated by the Hospital for Special Surgery (HSS) scoring system, and knee mobility was assessed by the Lysholm scoring system at the final follow-up^21,28^.

In this study, more than three bone fragments on the axial position of the CT scans were identified as comminuted fractures. The tibial plateau angle (TPA), medial posterior slope angle (PAM), plateau widening and step-off were measured by a surgeon with more than 10 years of experience. At the axial CT level, the fracture was defined as a coronary fracture when the fracture line was at -45° to 30° relative to the posterior femoral condylar axis^22,29^. Postoperative X-ray film showed that an intra-articular step-off of 2 mm or more, plateau widening of 5 mm or more, TPA ≥ 95°/ ≤ 80°, or PAM ≥ 15°/ < − 5° were defined as malreduction. Secondary loss of reduction was defined as intra-articular step-off greater than 3 mm, plateau widening was greater than 5 mm, and malalignment increased 5° at the final follow-up compared to immediate postoperative radiographs^1,8,30^. In addition, at least three cortical healings found on radiology were defined as fracture healing. Subcutaneous tissues infections were defined as superficial, and any infections requiring surgical debridement were defined as deep.

### Statistical analysis

All statistical data in our study were analysed using IBM Statistical package for Social Sciences, IBM SPSS version 26.0 (IBM Armonk, New York, USA, https://www.ibm.com/products/spss-statistics). Continuous variables are presented as the mean values and standard deviation (SD) and were determined using independent samples Student’s t-tests or Mann–Whitney *U* tests depending on whether the values of the variable was normally distributed. Categorical variables are expressed as numbers and percentages (%) and were determined using the chi-square test or Fisher’s test, as appropriate. In addition, continuous data with skewed distributions were categorized by reference values. A significance level was set at *P* < 0.05.

## Conclusion

For patients with bicondylar TPFs, dual plating combined with compressing bolt fixation could reduce the secondary loss of reduction rate and showed a better functional outcome than single dual plating fixation. Furthermore, the use of compression bolts did not increase the mean time of operation, mean intraoperative blood loss, or postoperative complications. A prospective randomized controlled trial with a large number of patients and longer follow-up are needed in the future to confirm the therapeutic traits of dual plating combined with compressing bolts.
